# Swine acute diarrhea syndrome coronavirus nsp5 induces apoptosis by targeting GATA zinc finger domain-containing protein 2A (GATAD2A/p66α)

**DOI:** 10.1128/mbio.00785-26

**Published:** 2026-06-15

**Authors:** Haixin Huang, Xin Wang, Yan Qin, Yuying Li, Jiahan Gang, Xinyu Zhang, Lulu Xie, Yimin Zhou, Qiaoqiong Wang, Wei Chen, Yuanze Sun, Zaijuan Zhang, Xianfu Ke, Tian Lan, Wenchao Sun

**Affiliations:** 1 School of Public Health, Beihua University 74568, Jilin City, Jilin Province, China; 2 College of Veterinary Medicine, Northwest A&F University 12469, Yangling, Shaanxi, China; 3 Wenzhou Key Laboratory for Virology and Immunology, Institute of Virology, Wenzhou University 26495, Wenzhou, Zhejiang, China; 4 Jilin Provincial Center for Disease Control and Prevention 665505 , Changchun, Jilin Province, China; 5 College of Animal Science and Veterinary Medicine, Heilongjiang Bayi Agricultural University 91625, Daqing, Harbin, China; 6 Zhejiang Key Laboratory of High-level Biosafety and Biomedical Transformation, Hangzhou Medical College 117839, Hangzhou, Zhejiang, China; Tsinghua University, Beijing, China

**Keywords:** SADS-CoV, nsp5, GATAD2A, apoptosis

## Abstract

Swine acute diarrhea syndrome coronavirus (SADS-CoV) causes severe enteric disease in neonatal piglets, resulting in substantial economic losses worldwide. The molecular mechanisms underlying SADS-CoV pathogenesis, particularly how viral proteins modulate host cell death, remain poorly understood. Here, we demonstrated that SADS-CoV infection induces apoptosis *in vitro,* and that non-structural protein 5 (nsp5) alone is sufficient to trigger apoptotic pathways. We identified GATA zinc finger domain-containing protein 2A (GATAD2A), a core component of the nucleosome remodeling and deacetylation (NuRD) complex, as a novel substrate of SADS-CoV nsp5. Mechanistically, nsp5-mediated cleavage of GATAD2A disrupts the recruitment of chromodomain helicase DNA-binding protein 4 (CHD4), leading to dysregulation of apoptosis-related gene transcription. Mutational analysis revealed that the catalytic residues His41 and Cys144 of nsp5 are essential for its pro-apoptotic activity and oxidative stress induction. Notably, GATAD2A functions as a negative regulator of nsp5-induced apoptosis. Furthermore, SADS-CoV nsp5 cleaves GATAD2A across multiple species, and cleavage at glutamine 531 (Q531) is conserved among nsp5 proteins from SARS-CoV, MERS-CoV, SARS-CoV-2, and PDCoV. Our findings uncover a previously unrecognized mechanism by which coronavirus nsp5 induces apoptosis through targeting GATAD2A.

**IMPORTANCE:**

Swine acute diarrhea syndrome coronavirus (SADS-CoV) is an emerging coronavirus that poses a significant threat to the global swine industry. The main protease nsp5 represents an attractive antiviral target due to its essential role in viral replication. While previous studies have focused on nsp5’s function in polyprotein processing and immune evasion, its role in regulating host cell death remains largely unexplored. Here, we report the first evidence that SADS-CoV nsp5 induces apoptosis by cleaving GATAD2A, a key component of the NuRD complex. Strikingly, GATAD2A cleavage at Q531 represents a conserved strategy employed by diverse coronaviruses, including SARS-CoV-2, to manipulate host cell fate. This study reveals a novel mechanism of coronavirus pathogenesis and identifies GATAD2A as a potential therapeutic target for broad-spectrum coronavirus intervention.

## INTRODUCTION

A novel coronavirus, swine acute diarrhea syndrome coronavirus (SADS-CoV), belongs to the genus *Alphacoronavirus* of the family *Coronaviridae* (1). It is an enveloped single-stranded positive-sense RNA virus characterized by a helical symmetry core and a distinctive crown-like morphology (2). It was first detected in Guangdong, China, in 2017 and subsequently spread to neighboring Fujian (2018), Jiangxi (2018), Guangxi (2021), and Henan (2023), causing significant economic losses to the pig farming industry (1, 3–5). Co-evolutionary analysis revealed that HKU2 might have accomplished host switching via the SADS-related coronavirus, which was isolated from the genus *Rhinolophus* (6). Previous research has synthesized a panel of contiguous cDNAs spanning the entire SADS-CoV genome that can replicate in human liver cells (Huh7.5), intestinal cells (CaCo2), gastrointestinal cells (ST-INT), and colorectal tumor cells (HRT) (7). These results indicate a potential risk of zoonotic disease transmission.

Among the non-structural proteins (nsps), nsp5, also known as 3C-like protease (3CL^pro^), plays a critical role in processing the viral polyprotein to generate mature non-structural proteins and typically consists of 303 amino acid residues (8–11). Nsp5 of coronaviruses features a highly conserved catalytic active site, with the S2 substrate-binding pocket exhibiting exceptional conservation across the genus and cooperating with adjacent domains to determine substrate specificity (12, 13). In respiratory epithelial cells, the SUD2-Nsp5 complex of severe acute respiratory syndrome coronavirus 2 (SARS-CoV-2) promoted BCL2-mediated apoptosis in a G4-dependent manner (14). The 3C-like protease nsp5 encoded by various coronaviruses exerts immunomodulatory effects through proteolytic targeting of host proteins. In porcine hosts, coronavirus nsp5 cleaves GSDMD at Q193, generating truncated fragments that cannot initiate pyroptosis and consequently lose the ability to restrain porcine epidemic diarrhea virus (PEDV) replication (15). PDCoV nsp5 targets a different host factor, POLDIP3, cleaving it at Q176 to destroy its antiviral function and promote viral replication (16). She et al. showed that SADS-CoV nsp5 proteolytically depletes IKKε, blocking its phosphorylation and thereby crippling the type I interferon response (17).

Apoptosis plays a central role in the host’s defense against viral infections (18–20). In response, pseudorabies virus (PRV) and Bombyx mori nucleopolyhedrovirus (BmNPV) have evolved diverse mechanisms to modulate apoptotic pathways and thereby escape the host’s antiviral immune responses (18, 19, 21). The E3 ubiquitin ligase TRIM7 has been reported to conjugate the SARS-CoV-2 membrane protein, thereby restricting both apoptotic cell death and viral propagation (22). Matrine exhibits antiviral activities against PEDV by directly targeting the spike protein of the virus and inducing apoptosis via the MAPK signaling pathway (23). The SADS-CoV accessory protein NS7a facilitates viral spread by triggering apoptosis, which, in turn, dampens type III interferon responses (24). In contrast, PEDV hijacks mitophagy as a pro-viral strategy, blocking apoptotic pathways and attenuating JAK/STAT1-mediated antiviral signaling (25).

The GATAD2 family, comprising GATAD2A and GATAD2B (formerly designated as p66α and p66β), serves as an integral constituent of the nucleosome remodeling and deacetylation (NuRD) machinery, alongside CHD3/4 chromodomain helicases (26). Within this multisubunit assembly, GATA zinc finger domain-containing 2A (GATAD2A) associates with histone deacetylases HDAC1/2, ATP-dependent chromatin remodelers, histone chaperones, and DNA-binding factors to coordinate chromatin-based transcriptional regulation (26–28). Viruses can exploit NuRD subunits for chromatin hijacking; conversely, disruption of components such as MTA2 or CHD helicases has been shown to antagonize viral replication (29). Notably, PDCoV nsp5 proteolytically processes HDAC2, thereby abrogating its antiviral function (28). The related GATAD2B-NuRD assembly governs homologous recombination-mediated DNA repair through chromatin architecture maintenance, and its loss provokes aberrant chromatin relaxation coupled with excessive DNA end processing, ultimately compromising genomic integrity and activating apoptotic cascades (30). DNA damage-induced PARP activation further links to apoptotic execution, as PARP serves as a caspase-3 proteolytic target (31, 32).

In our study, we confirmed that GATAD2A can inhibit the apoptosis induced by SADS-CoV and nsp5, but its effect on coronaviruses is still unclear. Our findings establish that SADS-CoV nsp5 induces host cell apoptosis through proteolytic targeting of GATAD2A. Notably, this GATAD2A-cleaving capability extends beyond SADS-CoV, as we observed comparable protease activity among nsp5 orthologs from MERS-CoV, SARS-CoV, and SARS-CoV-2. These results contribute new mechanistic insights into nsp5-mediated pathogenesis across coronavirus lineages.

## RESULTS

### SADS-CoV nsp5 triggers apoptosis

**Fig 1:**
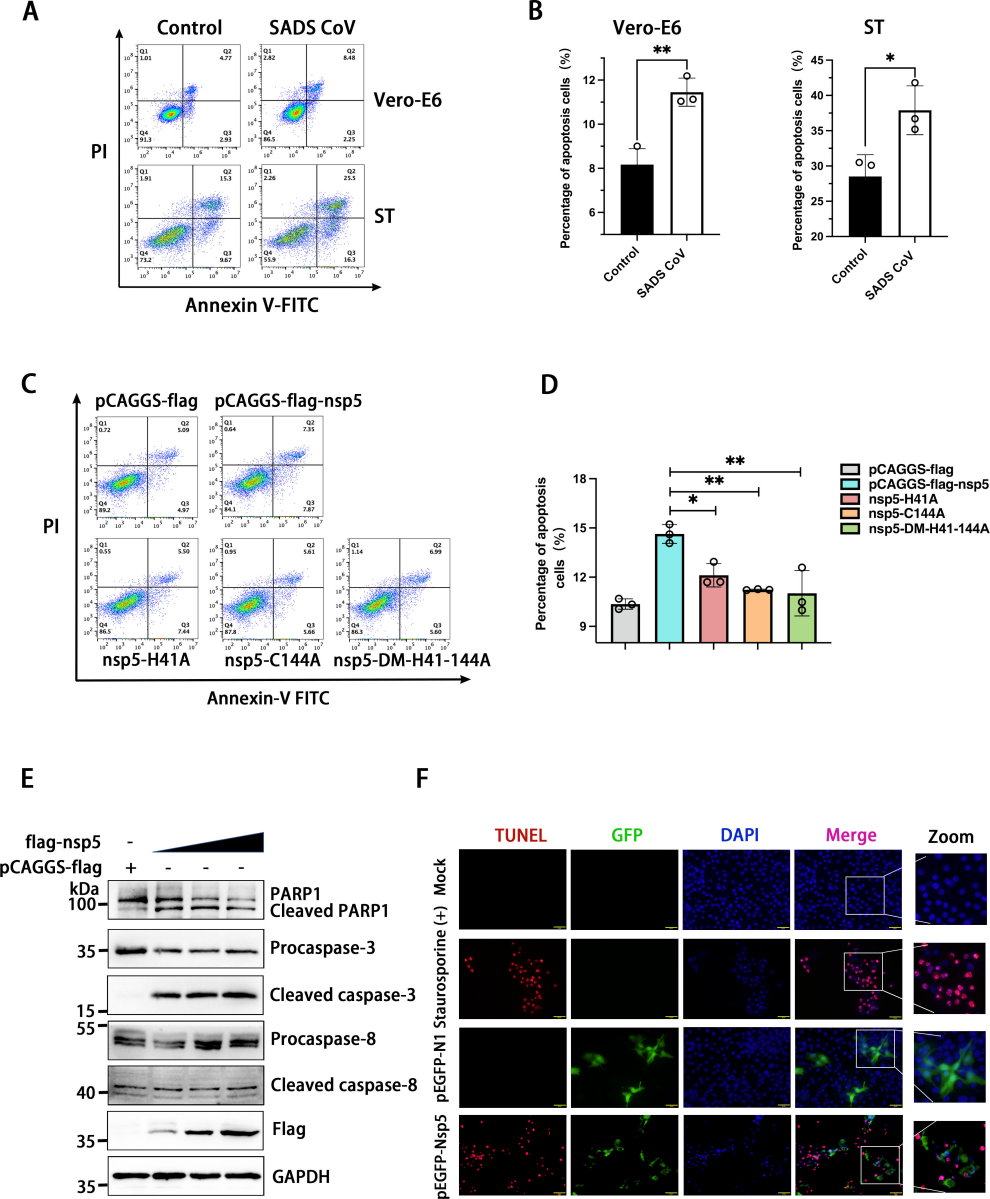
SADS-CoV nsp5 induces apoptosis in HEK 293T cells. (**A**) Cell death analysis by flow cytometry with dual Annexin V-PI cell labeling. SADS-CoV-infected Vero-E6 and ST cells collected at 24 h were subjected to dual Annexin V and PI labeling and analyzed by FlowJo v10. Lower-left quadrants represent intact cells (Annexin V negative/PI negative); lower-right quadrants represent early apoptotic cells (Annexin V positive/PI negative); upper-right quadrants indicate late apoptotic and/or necrotic cells (Annexin V positive/PI positive); and upper-left quadrants indicate necrotic cells (Annexin V negative/PI positive). (**B**) Percentage of Annexin V-negative and PI-positive cells in uninfected and SADS-CoV-infected Vero-E6 and ST cells. (**C**) HEK-293T cells were transfected with pCAGGS-flag empty vector, pCAGGS-flag-nsp5 expressing plasmid, mutant expressing plasmids H41A, C144A, and H41-144A, respectively, and collected at 36 h after transfection. Then, the cells were subjected to dual Annexin V and PI labeling and analyzed by FACS. (**D**) Percentage of apoptosis of HEK-293T cells transfected with pCAGGS-flag, pCAGGS-flag-nsp5, mutant expressing plasmids H41A, C144A, and H41-144A. (**E**) SADS-CoV nsp5 activates caspase-8, -3, and cleaved PARP in HEK-293T cells. (**F**) TUNEL labeling of pEGFP-N1-Nsp5 transfected Vero-E6 cells. Mock-transfected pEGFP-N1 empty vector, staurosporine-treated (10 µM) cells, and pEGFP-Nsp5-transfected cells fixed at 24 h were labeled with TUNEL (red) and sequentially stained with an anti-GFP antibody (green). Cells were counterstained with 4′,6-diamidino-2-phenylindole (DAPI), and photomicrographs of TUNEL labeling and nsp5 protein staining in virus-infected cells were obtained using a confocal microscope. All data are reported as the means ± SDs. For all experiments, *P < 0.05 and **P < 0.01 were considered to indicate statistical significance.

SADS-CoV infection of Vero-E6 cells resulted in significantly increased viral replication at 12, 24, and 36 h post-infection, as demonstrated by RT-qPCR of N gene copies and N protein detection (Fig. S1A through D). To investigate SADS-CoV-induced apoptosis, Vero-E6 cells were infected with SADS-CoV for 36 h and analyzed by Annexin V-FITC staining and flow cytometry. SADS-CoV infection induced significantly higher levels of apoptosis in Vero-E6 and ST cells compared with uninfected controls (Fig. 1A and B). Moreover, SADS-CoV triggered apoptosis in both cell lines in a dose-dependent manner across a range of MOIs, with maximal apoptotic responses observed at an MOI of 1 (Fig. S2A and D). The SADS-CoV nsp5 protease depends on catalytic residues histidine 41 (His 41) and cysteine 144 (Cys 144). In HEK-293T cells, wild-type nsp5 induced apoptosis, but mutants H41A, C144A, and DM-H41-C144A did not (Fig. 1C and D), demonstrating these residues are essential for nsp5 activity. Dose-dependent transfection of SADS-CoV nsp5 expression plasmid demonstrated that nsp5 induces apoptosis in a dose-dependent manner (Fig. 1E). TUNEL staining further confirmed enhanced DNA fragmentation in cells overexpressing nsp5 compared to empty vector controls, with staurosporine-treated cells as a positive control (Fig. 1F). These findings demonstrate that SADS-CoV nsp5 significantly induces apoptosis in host cells. We examined apoptosis-related protein expression in SADS-CoV-infected ST cells. Cleaved PARP1, caspase-3, and caspase-8 levels were significantly higher in infected cells than in controls. Time-course analysis showed progressive increases in these cleaved proteins with prolonged infection (Fig. S2E and F). Viral infection can induce reactive oxygen species (ROS) accumulation, leading to cellular damage and apoptosis. We assessed ROS production in cells transfected with SADS-CoV nsp5 or its mutants. Nsp5 overexpression significantly increased intracellular ROS, whereas His 41 and Cys 144 mutants showed reduced ROS induction (Fig. S2G and H). These data suggest that SADS-CoV induces apoptosis in host cells *in vitro*.

### GATAD2A positively regulates the type I IFN signaling pathway

**Fig 2:**
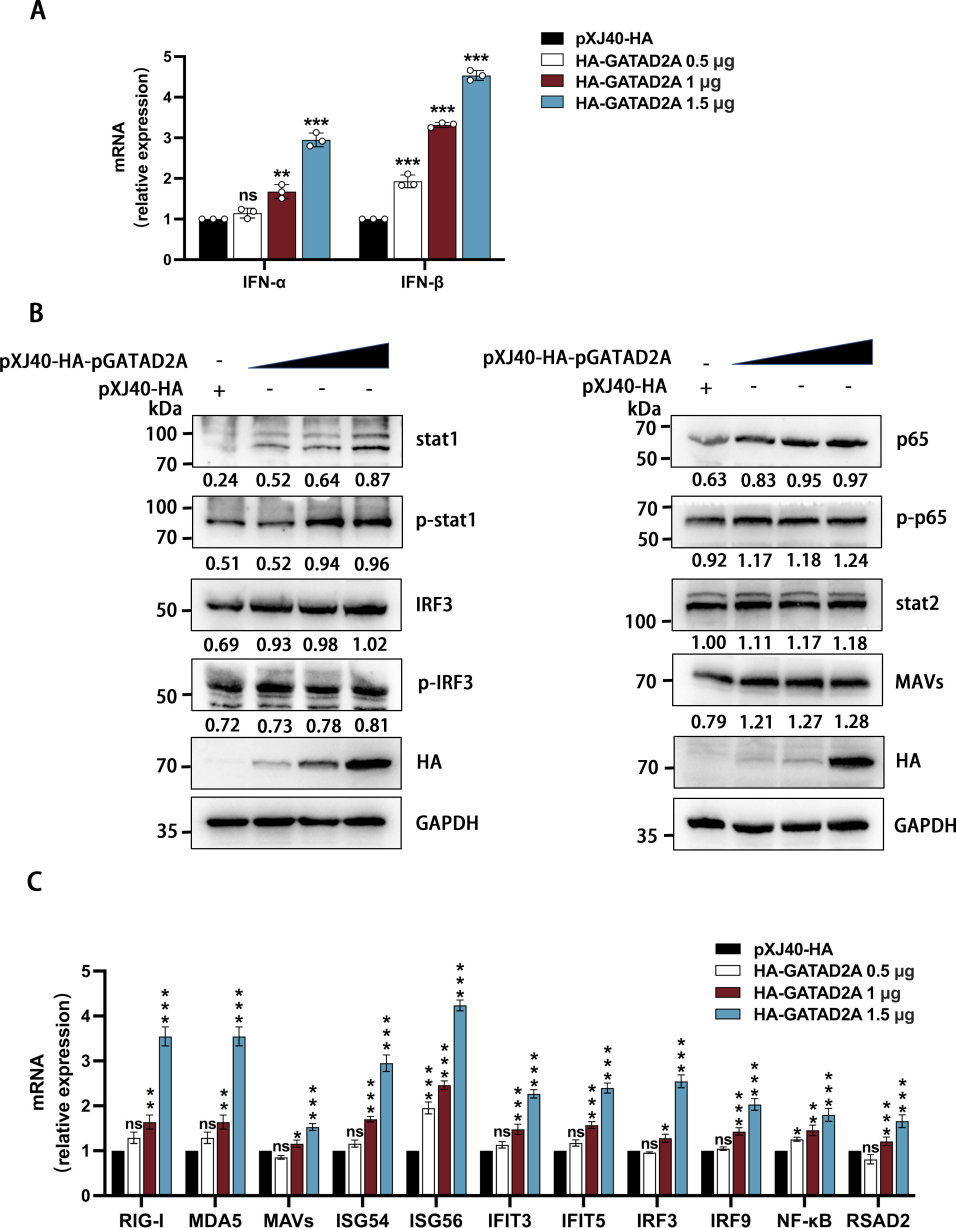
GATAD2A activates the type I IFN signaling pathway. (**A**) HEK-293T cells were transfected with 0.5 μg, 1 μg, or 1.5 μg of the pXJ40-HA-pGATAD2A. After 30 h, the cells were collected, and the IFN-α and IFN-β mRNA levels were measured by RT**-**qPCR. (**B**) pXJ40-HA-vector and 0.5 μg, 1 μg, or 1.5 μg of the pXJ40-HA-pGATAD2A were transfected into HEK-293T cells. After 30 h of transfection, the cells were collected and analyzed by Western blotting with anti-p65, anti-phospho-p65, anti-IRF3, anti-phospho-IRF3, anti-STAT1, anti-phospho-STAT1, anti-STAT2, anti-MAVS, and anti-HA and anti-GAPDH antibodies. Relative levels of p65, phospho-p65, IRF3, phospho-IRF3, STAT1, phospho-STAT1, STAT2, and MAVS were quantified and normalized to the GAPDH expression level using ImageJ. (**C**) HEK-293T cells were transfected with 0.5 μg, 1 μg, or 1.5 μg of the pXJ40-HA-pGATAD2A. After 30 h, the cells were collected, and the RIG-I, MDA5, MAVS, ISG54, ISG56, IFIT3, IFIT5, IRF3, IRF9, NF-κB, and RSAD2 mRNA levels were measured by RT-qPCR. All data are reported as the means ± SDs. For all experiments, *P < 0.05, **P < 0.01, and ***P < 0.001 were considered to indicate statistical significance. *ns*, nonsignificant difference.

To investigate the impact of GATAD2A on the IFN-I signaling pathway, increasing amounts of the pXJ40-HA-pGATAD2A plasmid were transfected into HEK-293T cells. The expression levels of key proteins in the IFN-I signaling pathway were analyzed 24 h post-transfection. As shown in Fig. 2A, overexpression of GATAD2A significantly increased the mRNA expression levels of IFN-α and IFN-β in a dose-dependent manner. The protein expression levels of key signaling molecules in the IFN-I signaling pathway were further analyzed. As shown in Fig. 2B, overexpression of GATAD2A increased the protein expression levels of IRF3, STAT1, STAT2, MAVS, and p65, as well as the phosphorylation levels of IRF3, STAT1, and p65. Next, the effect of GATAD2A on the interferon-stimulated genes (ISGs) of the IFN-I signaling pathway was detected. The results demonstrated that GATAD2A transfection significantly enhanced the mRNA expression levels of ISGs, including RIG-I, MDA5, MAVS, ISG54, ISG56, IFIT3, IFIT5, IRF3, IRF9, NF-κB, and RSAD2 (Fig. 2C).

### GATAD2A inhibits SADS-CoV nsp5-induced apoptosis

**Fig 3:**
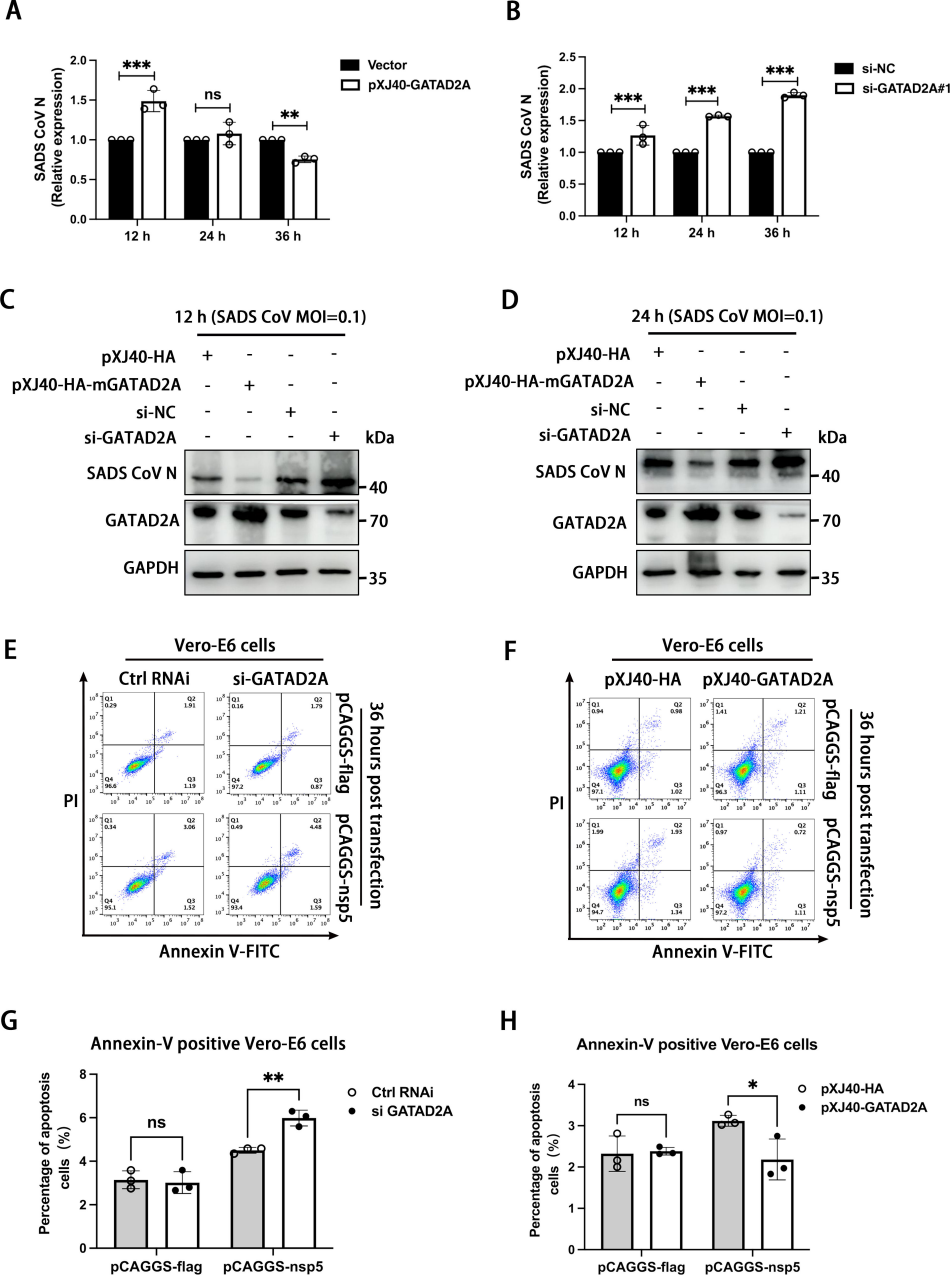
GATAD2A inhibits SADS-CoV nsp5-induced apoptosis. (**A**) Vero-E6 cells cultured in 12-well plates were transfected with pXJ40-HA-mGATAD2A expression plasmids. After 24 h, cells were infected with SADS-CoV for 12 h, 24 h, and 36 h. And then, total RNA, lysed products, and supernatants were collected separately. The mRNA levels of SADS-CoV N were evaluated by RT-qPCR. (**B**) GATAD2A siRNA and negative control siRNA were transfected into Vero-E6 cells. After 24 h, cells were infected with SADS-CoV for 12 h, 24 h, and 36 h. And then, total RNA, lysed products, and supernatants were collected separately. The mRNA levels of SADS-CoV N were evaluated by RT-qPCR. (**C and D**) pXJ40-HA-vector, pXJ40-HA-mGATAD2A, GATAD2A siRNA, and negative control siRNA were transfected into Vero-E6 cells. After 12 h of transfection, the cells were infected with SADS-CoV (MOI = 0.1) for 12 h, and then 24 h later, the cells were harvested for Western blot analysis. (**E**) Vero-E6 cells were transfected with negative control siRNA, GATAD2A siRNA, pCAGGS-flag, and pCAGGS-flag-nsp5, collected at 36 h after transfection. Then, the cells were assessed by flow cytometry assay. (**F**) Vero-E6 cells were transfected with pXJ40-HA, pXJ40-HA-mGATAD2A, pCAGGS-flag, and pCAGGS-flag-nsp5, respectively, and collected at 36 h after transfection. Then cells were subjected to dual Annexin V and PI labeling and analyzed by FACS. (**G**) Percentage of apoptosis analysis with pCAGGS-flag, pCAGGS-flag-nsp5, negative control siRNA, and GATAD2A siRNA. (**H**) Percentage of apoptosis analysis with pCAGGS-flag, pCAGGS-flag-nsp5, negative control pXJ40-HA, and pXJ40-HA-mGATAD2A. All data are reported as means ± SDs. For all experiments, *P < 0.05, **P < 0.01, and ***P < 0.001 were considered to indicate statistical significance. *ns*, nonsignificant difference.

To further elucidate the functional mechanism of GATAD2A in the replication of SADS-CoV, the GATAD2A gene was cloned into the pXJ40-HA expression vector. Following 24 h of transfection with pXJ40-HA-mGATAD2A in Vero-E6 cells, SADS-CoV was inoculated, and viral replication was assessed by measuring the expression level of the SADS-CoV N gene. As shown in Fig. 3A, the inhibitory effect of GATAD2A on viral replication was found to be more pronounced with prolonged viral infection time. To further investigate this regulatory effect, GATAD2A-specific siRNA was transfected into Vero-E6 cells, followed by SADS-CoV inoculation after 24 h. Cell samples were collected at 12, 24, and 36 h post-infection to assess viral gene replication levels. The results demonstrated that GATAD2A knockdown significantly enhanced viral replication (Fig. 3B). At 12 and 24 h post-inoculation with SADS-CoV (MOI = 0.1), samples were collected to assess the expression levels of viral proteins. The results demonstrated that GATAD2A overexpression significantly suppressed the expression of the SADS-CoV N protein at 12 h and 24 h post-viral infection, whereas knockdown of GATAD2A led to a notable enhancement in SADS-CoV N protein expression (Fig. 3C and D). Previous studies have demonstrated that GATAD2A inhibits SADS-CoV replication and promotes activation of the IFN-I signaling pathway. However, the mechanism by which GATAD2A enhances IFN-I signaling to exert its antiviral effects against SADS-CoV remains to be elucidated. Several studies have reported the existence of a regulatory crosstalk between apoptosis and the IFN signaling pathway. However, it remains to be determined whether GATAD2A inhibits apoptosis and thereby promotes IFN expression. Further infection of Vero-E6 cells with SADS-CoV was conducted, and the level of cell apoptosis was detected 24 h after infection. The results showed that the proportion of cell apoptosis induced by SADS-CoV was significantly reduced after overexpression of GATAD2A (Fig. S3A and C). Following GATAD2A knockdown, the proportion of apoptosis induced by SADS-CoV was significantly increased (Fig. S3B and D). Earlier research has demonstrated that the nsp5 of SADS-CoV is capable of triggering apoptotic processes in host cells. To evaluate the regulatory role of GATAD2A in apoptosis, SADS-CoV nsp5 was transfected into Vero-E6 cells to trigger apoptotic signaling, while GATAD2A was either knocked down or overexpressed. The effect of GATAD2A modulation on SADS-CoV nsp5-induced apoptosis was subsequently assessed. Apoptosis levels were analyzed by flow cytometry 36 h post-transfection. The results revealed that GATAD2A knockdown significantly increased the proportion of apoptosis (Fig. 3E and G). In contrast, GATAD2A overexpression significantly attenuated SADS-CoV nsp5-induced apoptosis (Fig. 3F and H).

### SADS-CoV nsp5 interacts with GATAD2A

**Fig 4:**
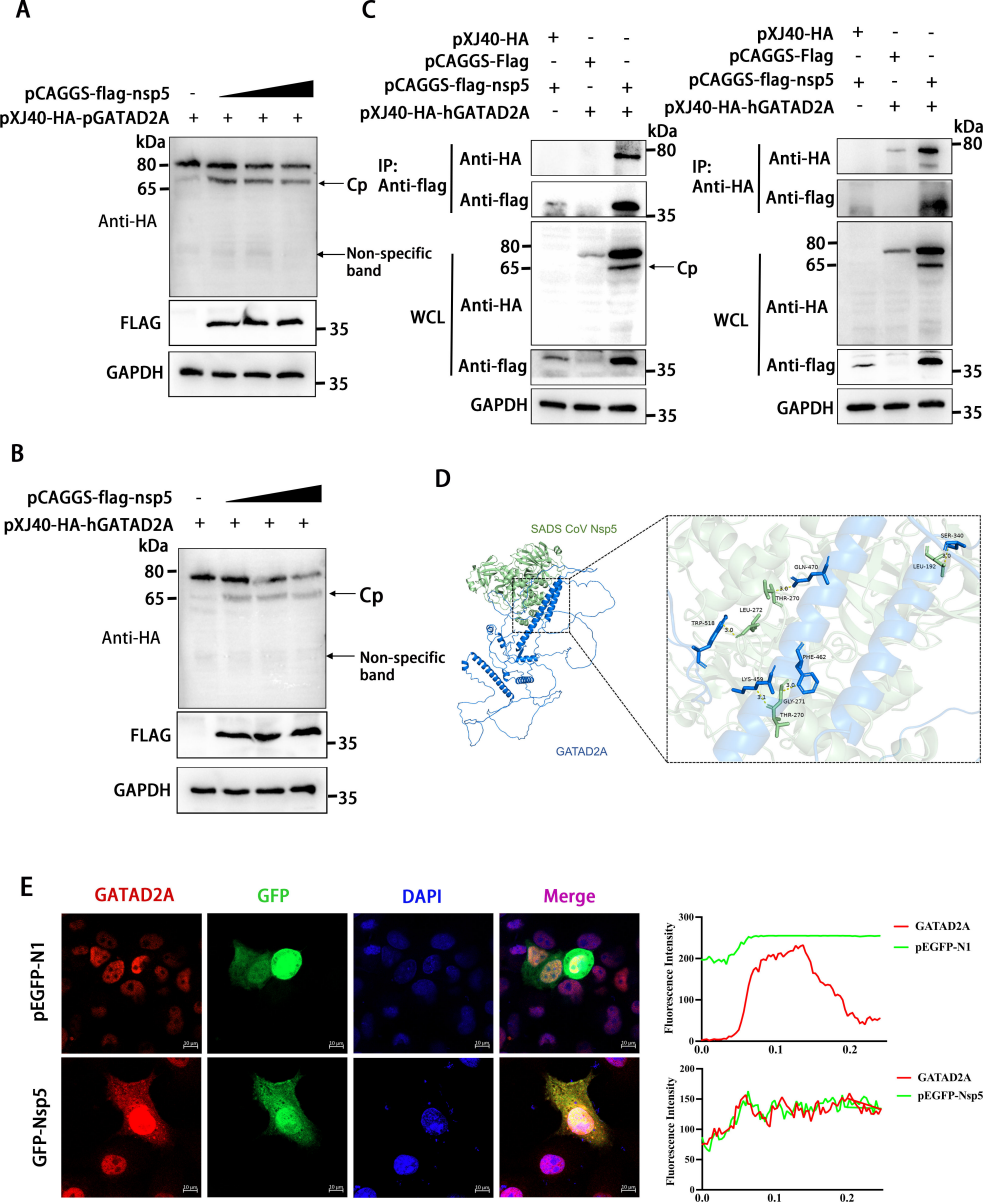
SADS-CoV nsp5 targets GATAD2A for cleavage. (**A**) HEK-293T cells were co-transfected with pXJ40-HA-pGATAD2A and various amounts of pCAGGS-flag-nsp5. After 30 h, the cells were lysed for Western blotting. The cleavage products are labeled “Cp.” (**B**) HEK-293T cells were co-transfected with pXJ40-HA-hGATAD2A and various amounts of pCAGGS-flag-nsp5. After 30 h, the cells were lysed for Western blotting. (**C**) HEK-293T cells were transfected with expression constructs encoding pCAGGS-flag-nsp5 and pXJ40-HA-hGATAD2A. The cells were lysed 30 h after transfection and subjected to immunoprecipitation with an anti-FLAG antibody or an anti-HA antibody. The whole-cell lysates (WCLs) and immunoprecipitation (IP) complexes were analyzed by immunoblotting (IB) using anti-FLAG, anti-HA, or anti-GAPDH antibodies. (**D**) The GATAD2A protein is represented as a blue cartoon model, the nsp5 protein is shown as a green cartoon model. When focusing on the binding region, the binding site is then shown as a presentation of the protein to which it belongs. (**E**) ST cells were transfected with SADS-CoV nsp5 and pEGFP-N1. After 24 h, the cells were fixed and then stained with a rabbit monoclonal antibody against GATAD2A and a mouse anti-GFP Tag antibody prior to incubation with an Alexa Fluor 488-conjugated goat anti-mouse IgG antibody (green) or the Alexa Fluor 594-conjugated goat anti-rabbit IgG antibody (red). Nuclei were stained with DAPI (blue).

Previous studies have demonstrated that GATAD2A effectively suppresses apoptosis induced by SADS-CoV and its nsp5 protein. Given that both SADS-CoV infection and nsp5 protein transfection significantly induce apoptotic responses, it is hypothesized that the SADS-CoV nsp5 protein may interact with GATAD2A to attenuate its functional activity, thereby promoting apoptosis. To investigate the impact of SADS-CoV nsp5 on GATAD2A expression, the SADS-CoV nsp5 plasmid was co-transfected with the pXJ40-HA-pGATAD2A plasmid into HEK-293T cells, followed by assessment of GATAD2A expression levels. As shown in Fig. 4A, the expression of SADS-CoV nsp5 markedly suppressed the expression of porcine GATAD2A, with a potential cleavage band being observed. Furthermore, SADS-CoV nsp5 and human GATAD2A (pXJ40-HA-hGATAD2A) were co-transfected into HEK-293T cells to detect the effect of SADS-CoV nsp5 on the expression and cleavage of GATAD2A (Fig. 4B). These findings suggest that SADS-CoV nsp5 can cleave GATAD2A across multiple species and markedly suppresses its expression. To further confirm the potential interaction between SADS-CoV nsp5 and GATAD2A, co-immunoprecipitation assays combined with molecular docking analysis were conducted. The results demonstrated that SADS-CoV nsp5 can interact with GATAD2A (Fig. 4C). Molecular docking analysis predicted that SADS-CoV nsp5 can interact with GATAD2A (Fig. 4D). The SADS-CoV nsp5 gene was further cloned into the pEGFP-N1 empty vector to generate a GFP-tagged SADS-CoV nsp5 expression plasmid. Laser scanning confocal microscopy combined with immunofluorescence staining was employed to assess the co-localization of SADS-CoV nsp5 and GATAD2A. As shown in Fig. 4E, pEGFP-nsp5 exhibited clear co-localization with GATAD2A, whereas the pEGFP-N1 empty vector showed no detectable co-localization with GATAD2A. These findings demonstrate that SADS-CoV nsp5 can directly interact with GATAD2A to suppress its expression and mediate its cleavage.

### SADS-CoV nsp5 cleaves GATAD2A at Gln-531

**Fig 5:**
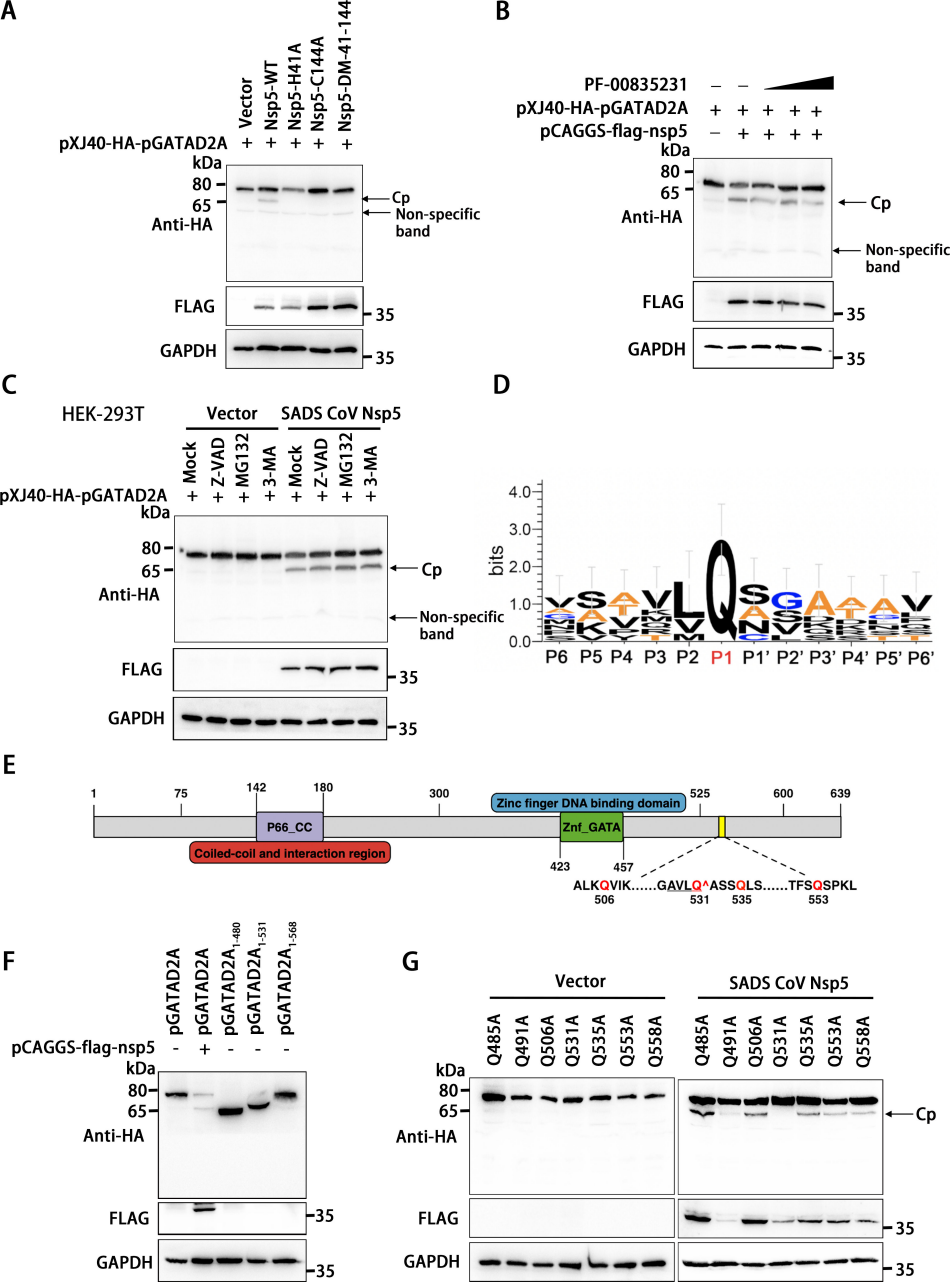
SADS-CoV nsp5 recognizes and cleaves GATAD2A at residue Q531. (**A**) HEK-293T cells were co-transfected with plasmids expressing the wild-type SADS-CoV nsp5 or one of the protease-defective mutants (C144A and H41A) and the pXJ40-HA-pGATAD2A. After 30 h, the cells were lysed, and supernatants were used for Western blotting. (**B**) HEK-293T cells were co-transfected with pXJ-HA-pGATAD2A and pCAGGS-flag-nsp5. After 24 h, the cells were mock-treated or treated with PF-00835231 (10 μM). After 12 h, the cells were lysed for Western blotting. (**C**) HEK-293T cells were co-transfected with plasmids expressing the wild-type SADS-CoV nsp5 and pXJ40-HA-pGATAD2A. After 24 h, the cells were treated with MG132 (10 μM), Z-VAD-FMK (25 μM), or 3-MA (10 μM) for 12 h. Cell lysates were prepared and analyzed by Western blotting. (**D**) Sequence logo of the polyprotein junctions that are cleaved by SADS-CoV nsp5. An amino acid sequence logo of the substrate was generated by WebLogo version 3. (**E**) Schematic representation of porcine GATAD2A and its mutation sites. (**F**) HEK-293T cells were transfected with expression constructs encoding pXJ40-HA-pGATAD2A, the pGATAD2A _1–480_ truncated mutant, pGATAD2A _1–531_ truncated mutant, and pGATAD2A_1–568_ truncated mutant and were collected after 30 h for Western blotting. (**G**) HEK-293T cells were co-transfected with the pCAGGS-flag and pCAGGS-flag-nsp5 along with expression constructs encoding wild-type pGATAD2A or pGATAD2A mutants (pGATAD2A-Q485A, pGATAD2A-Q491A, pGATAD2A-Q506A, pGATAD2A-Q531A, pGATAD2A-Q553A, and pGATAD2A-Q558A). The cells were then lysed after 30 h and analyzed by Western blotting.

Previous studies have identified that the His 41 and Cys 144 residues within SADS-CoV nsp5 constitute its protease active sites. Notably, SADS-CoV nsp5 has been shown to significantly suppress the expression of GATAD2A and mediate its cleavage. To verify that SADS-CoV nsp5 utilizes its protease activity to cleave GATAD2A, SADS-CoV nsp5 mutants at positions 41 and 144 amino acids were co-transfected with GATAD2A. The cleavage of GATAD2A by SADS-CoV nsp5 was detected 24 h after transfection. As shown in Fig. 5A, mutation of the 41st and 144th amino acid residues in SADS-CoV nsp5 resulted in the loss of its proteolytic activity against GATAD2A. Subsequently, SADS-CoV nsp5 and GATAD2A co-transfected cells were treated with PF-00835231, a specific inhibitor of coronavirus nsp5, and the proteolytic activity of SADS-CoV nsp5 on GATAD2A was assessed 24 h post-transfection. The experimental results demonstrated that PF-00835231 treatment markedly reduced the proteolytic activity of SADS-CoV nsp5 against GATAD2A in a dose-dependent manner (Fig. 5B). To verify whether the cleavage of GATAD2A by SADS-CoV nsp5 solely depends on its own protease activity, nsp5 and GATAD2A co-transfected cells were further treated with three inhibitors (Z-VAD, MG132, and 3-MA). The cleavage activity of nsp5 on GATAD2A was detected 24 h after transfection. As shown in Fig. 5C, none of the three inhibitors exhibited inhibitory effects on the cleavage of GATAD2A mediated by nsp5. To further elucidate the specific cleavage site on GATAD2A recognized by nsp5, we analyzed the substrate recognition characteristics of coronavirus nsp5. Based on these characteristics and the predicted size of GATAD2A cleavage products, we generated schematic diagrams of potential cleavage sites (Fig. 5D and E). Truncated expression plasmids encoding GATAD2A_1–480_, GATAD2A_1–531_, and GATAD2A_1–568_ were generated based on the predictive analysis and subsequently compared with the full-length GATAD2A to define the cleavage site region (Fig. 5F). Based on the range of cleavage sites, the glutamine amino acid mutants at positions 485, 491, 506, 531, 535, 553, and 558 of GATAD2A were constructed. The mutant expression plasmids and nsp5 expression plasmids were co-transfected into HEK-293T cells to detect the cleavage effect of nsp5 on GATAD2A mutants. Nsp5 specifically recognizes and cleaves GATAD2A at the glutamine residue located at position 531 through its intrinsic protease activity (Fig. 5G).

### The efficiency of nsp5-mediated cleavage of GATAD2A differs across coronaviruses

**Fig 6:**
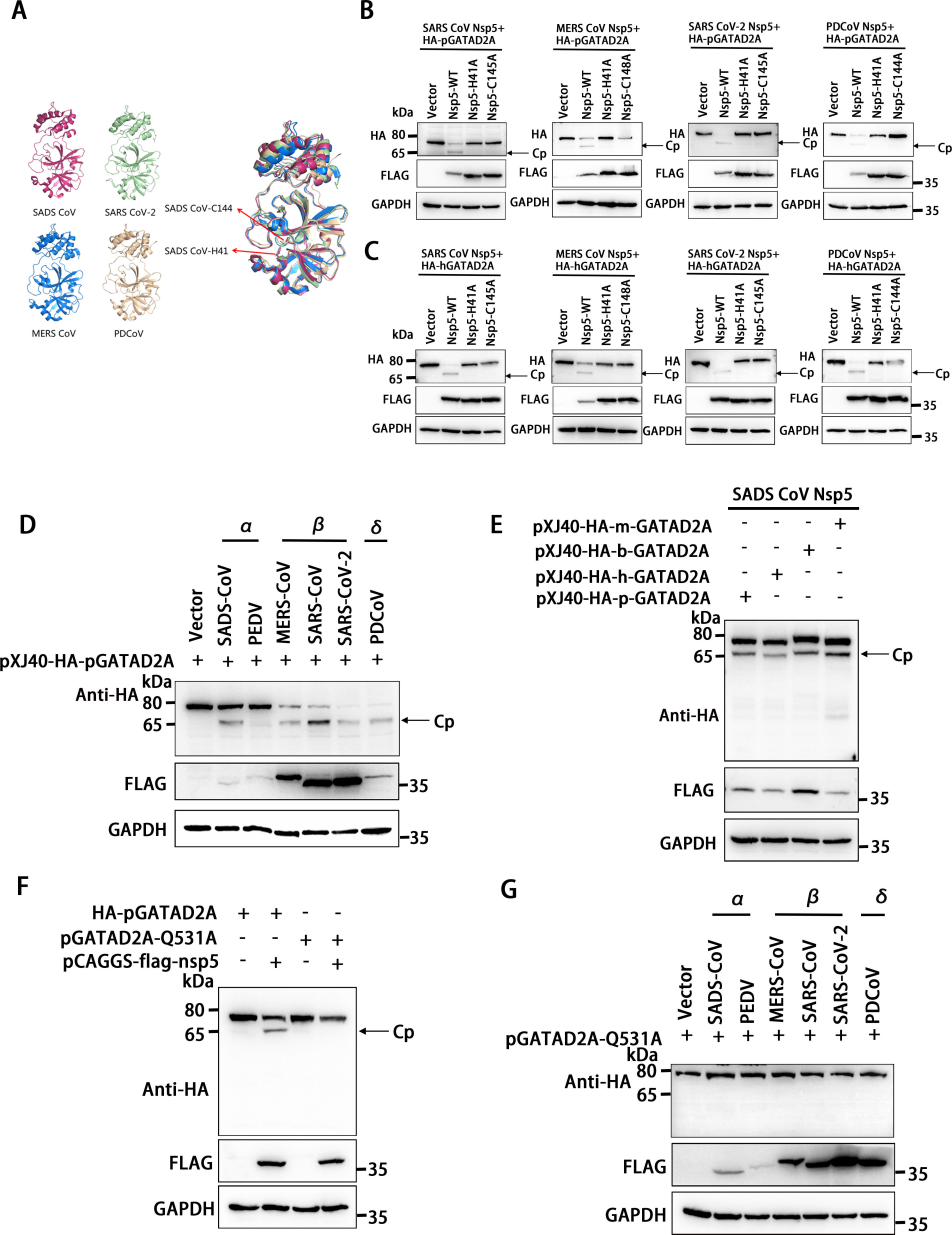
The efficiency of Nsp5-mediated cleavage of GATAD2A differs across coronaviruses. (**A**) Structural alignment of the conserved residues His41 and Cys144 in CoV nsp5s. The homology model of SADS-CoV nsp5 was generated based on the crystal structures of PDCoV nsp5 by the software Modeller, version 9.14. The 3D structures of PDCoV nsp5 (PDB accession number 7WKU), SARS-CoV nsp5 (PDB accession number 2Q6G), SARS-CoV-2 nsp5 (PDB accession number 8WSK), and MERS nsp5 (PDB accession number 9BOO) were obtained from the Protein Data Bank. Red arrows indicate conserved enzymatic proteolysis residues. (**B and C**) HEK-293T cells were co-transfected with plasmids expressing the wild-type SARS-CoV nsp5 or one of the protease-defective mutants (C145A and H41A), wild-type MERS-CoV nsp5 or one of the protease-defective mutants (C148A and H41A), wild-type SARS-CoV-2 nsp5 or one of the protease-defective mutants (C145A and H41A), wild-type SARS-CoV-2 nsp5 or one of the protease-defective mutants (C144A and H41A), and the pXJ40-HA-pGATAD2A or pXJ40-HA-hGATAD2A. After 30 h, the cells were lysed, and supernatants were used for Western blotting. (**D**) HEK-293T cells were cultured in 12-well plates and co-transfected with pXJ40-HA-pGATAD2A and a plasmid encoding the nsp5 of SADS-CoV, SARS-CoV-2, SARS-CoV, MERS-CoV, PEDV, or PDCoV. After 30 h, the cells were lysed and analyzed by Western blotting. (**E**) HEK-293T cells were co-transfected with SADS-CoV nsp5 and pXJ-HA-b-GATAD2A (bovine), pXJ-HA-m-GATAD2A (monkey), pXJ-HA-h-GATAD2A (human), and pXJ-HA-p-GATAD2A (porcine). After 30 h, the cells were lysed and analyzed by Western blotting. (**F**) HEK-293T cells were co-transfected with the SADS-CoV nsp5 expression plasmid along with wild-type GATAD2A or pGATAD2A-Q531A and collected after 30 h for Western blotting. (**G**) HEK-293T cells were co-transfected with the SADS-CoV, PEDV, MERS-CoV, SARS-CoV, SARS-CoV-2, and PDCoV nsp5 expression plasmid along with pGATAD2A-Q531A and collected after 30 h for Western blotting.

To further investigate whether the cleavage of GATAD2A by SADS-CoV is evolutionarily conserved across different coronaviruses, we performed a comparative analysis of the protein structures of SADS-CoV, MERS-CoV, SARS-CoV-2, and PDCoV, followed by homology modeling (Fig. 6A). The nsp5 mutant plasmids were respectively co-transfected with porcine or human GATAD2A (pGATAD2A and hGATAD2A) into HEK-293T cells. The cleavage of different species of GATAD2A by different coronavirus nsp5 and its mutants was analyzed by Western blotting. As shown in Fig. 6B and C, the nsp5 proteins from multiple coronaviruses can cleave GATAD2A. However, this proteolytic activity is abolished in mutants carrying substitutions at residues His 41 and Cys 145. These findings demonstrate that the protease active sites of coronavirus nsp5 proteins universally rely on histidine at position 41 and cysteine at position 145 for catalytic function. Furthermore, bovine and monkey sources of GATAD2A (bGATAD2A and mGATAD2A) were constructed and co-transfected with SADS-CoV nsp5. As shown in Fig. 6D, the nsp5 of different coronavirus genera can all target GATAD2A for cleavage. As shown in Fig. 6E, SADS-CoV nsp5 can cleave GATAD2A across multiple species, including porcine, human, bovine, and monkey origins. These findings demonstrate that GATAD2A serves as a conserved target for nsp5 proteins from diverse coronaviruses. To further validate the precision of the cleavage recognition site, expression plasmids encoding the GATAD2A glutamine 531 mutant and SADS-CoV nsp5 were transfected separately into cells. The cleavage activity of SADS-CoV nsp5 toward the GATAD2A mutant was subsequently assessed, yielding results consistent with previous studies (Fig. 6F). These findings demonstrate that SADS-CoV nsp5 specifically recognizes and cleaves GATAD2A at glutamine residue 531 in a protease activity-dependent manner. To further assess the conservation of this cleavage across coronaviruses, we examined whether nsp5 proteins from PEDV, MERS-CoV, SARS-CoV, SARS-CoV-2, and PDCoV also recognize and cleave GATAD2A at the identical glutamine 531 residue. As shown in Fig. 6G, PEDV nsp5, MERS-CoV nsp5, SARS-CoV nsp5, SARS-CoV-2 nsp5, and PDCoV nsp5 were co-transfected with GATAD2A with a glutamine 531 mutation, respectively, to detect their cleavage effect on GATAD2A. The results showed that the proteins of PEDV nsp5, MERS-CoV nsp5, SARS-CoV nsp5, SARS-CoV-2 nsp5, and PDCoV nsp5 could not cleave GATAD2A with a glutamine 531 mutation. Studies have shown that various coronaviruses can cleave GATAD2A, and the cleavage site is at the glutamine residue at position 531.

## DISCUSSION

SADS-CoV mainly causes acute diarrhea in piglets and is highly lethal, especially to newborn piglets (33). SADS-CoV has a wide range of host tissue tropism, and the furin-dependent cleavage of its spike protein and the utilization of TMPRSS13 may facilitate cross-species transmission (34, 35). Apoptosis, often referred to as programmed cell death, is a widely observed cellular process involved in eliminating unnecessary, damaged, or virally infected cells (36, 37). Upon stimulation by death ligands such as TRAIL, cells exhibit caspase-8 dimerization, proteolytic processing, and subsequent activation, leading to the direct cleavage and activation of the downstream effector caspase-3 (38). The initiation of caspase-3 indicates the onset of the apoptotic execution stage (39). The functional interaction between IFN and apoptosis constitutes a fundamental mechanism in host immune defense and presents a promising therapeutic target for disease intervention (40, 41). In our study, we confirmed that GATAD2A inhibits apoptosis induced by SADS-CoV and nsp5, and we further investigated the molecular mechanism.

Coronavirus nsp5 exerts a critical regulatory function in multiple signaling pathways, modulating the host immune response and viral replication through mechanisms involving pyroptosis (15), apoptosis (42), autophagy (43), regulation of the NF-κB signaling pathway (44), suppression of IFN signaling (45–47), and targeting of host tRNA modification enzymes (48). Studies have shown that the nsp5 of PEDV can induce apoptosis and disrupt mitochondrial membrane potential in a protease activity-dependent manner (42). The nsp5 and ORF6 of SARS-CoV-2 inhibit the activity of caspase-8 by acting on its large and small subunits, preventing the activation of apoptosis and pyroptosis. Nsp13 interacts with RIPK3 and interferes with its binding to ZBP1, thereby suppressing necroptosis triggered by ZBP1 (49). SADS-CoV nsp5 has been confirmed as an interferon antagonist that can indirectly affect apoptosis by suppressing the host immune response (47). In our study, we also confirmed that SADS-CoV can infect different host cells and induce apoptosis. Previous results indicated that the His41 and Cys144 residues of SADS-CoV nsp5 not only participate in polyprotein cleavage but also regulate the immune response by degrading the host protein DCP1A, thereby suppressing the production of IFN and inflammatory factors (47). Coronavirus infection significantly increases the level of reactive oxygen species (ROS) within cells, leading to oxidative stress. Excessive ROS can cause oxidative damage to lipids, proteins, and DNA, thereby resulting in tissue damage (50–52). In this study, we found that mutations at His 41 and Cys 144 in the nsp5 protein of SADS-CoV lead to a reduced capacity to induce cellular apoptosis and oxidative stress. Song et al. demonstrated that the endoplasmic reticulum (ER) protein stimulator of interferon genes (STING) inhibits the replication of coronaviruses by preventing the formation of double-membrane vesicles (DMVs) derived from the ER (53). Chu et al. reported that caspase-6 is a critical host factor that facilitates efficient coronavirus replication. Evidence indicates that caspase-6 mediates cleavage of the viral nucleocapsid protein, yielding fragments that function as interferon antagonists, thereby enhancing viral replication (54). Receptor transport protein 4 (RTP4), as a host-limiting factor, was observed to have significantly higher levels of ISGs in a mouse model of SARS-CoV-2, indicating that RTP4 can restrict coronavirus infection (55). Histone deacetylase 3 contributes to the antiviral innate immunity of macrophages by interacting with FOXK1 to regulate STAT1/2 transcription (56). Our research findings indicate that overexpression of GATAD2A reduces the replication of SADS-CoV. Furthermore, our investigation revealed that GATAD2A activates the type I interferon signaling pathway. Zhang et al. demonstrated that GATAD2A facilitates p53-dependent transcription by promoting p53 occupancy at target promoters, thereby upregulating apoptosis-associated genes. Genetic ablation of GATAD2A diminishes expression of p53-responsive targets, attenuates apoptotic capacity, and accelerates tumor progression in breast cancer models (57). Emerging evidence indicates that the pro-apoptotic potential of coronavirus nsp5 may be a conserved feature across different coronavirus species. Li et al. showed that the SARS-CoV-2 nsp3 SUD2 domain associates with nsp5 to form a ternary complex with heightened affinity for G-quadruplex motifs within the BCL-2 promoter region. This interaction diminishes BclII expression, thereby removing a key brake on apoptotic execution (14). Across multiple coronavirus species, SARS-CoV-2, MERS-CoV, PDCoV, and PEDV nsp5 proteolytically inactivates gasdermin D at residue Q193. The resulting cleavage products lack pore-forming capacity, which disables pyroptotic signaling and creates a cellular environment favorable to viral propagation (58). These findings suggest that coronavirus nsp5 may exhibit a dual regulatory role in cell death pathways, promoting apoptosis through interactions with host transcriptional regulators while inhibiting pyroptosis through direct proteolytic cleavage of key cell death executors. In our study, SADS-CoV nsp5 was shown to induce apoptosis, which is consistent with the pro-apoptotic function of nsp5 reported in other coronaviruses. Based on previous studies and our accumulated findings, we hypothesize that SADS-CoV modulates the antiviral response following infection, and that the nsp5 protein plays a critical role in facilitating virus-induced apoptosis, potentially through a mechanism involving GATAD2A. Moreover, our experimental results demonstrated that the nsp5 protein from SADS-CoV directly interacts with GATAD2A and cleaves GATAD2A to disrupt the innate immune pathways of the host and escape from host immune response.

NBR1, a selective autophagy receptor, restricts PDCoV replication. PDCoV nsp5 cleaves porcine NBR1 at Q353, disabling its autophagy function and antiviral activity (59). A parallel observation was made for SARS-CoV-2, where nsp5 cleaves p62 at Q354 to prevent autophagic clearance of the viral M protein (43). In our study, the nsp5 of SADS-CoV relies on its protease activity to cleave GATAD2A at the Q531 residue, and this cleavage can also be mediated by nsp5 proteins from multiple coronavirus species. Collectively, these results extend our understanding of coronavirus nsp5-mediated antagonism of host antiviral responses. Fragment screening studies of SARS-CoV-2 M^pro^ have identified multiple ligand-binding sites beyond the active site, suggesting complex allosteric regulation that may influence substrate turnover under conditions of enzyme excess (60, 61). Excess enzyme may perturb the dimer–monomer equilibrium or promote non-productive self-association, thereby paradoxically reducing the fraction of catalytically competent protease, a phenomenon supported by recent studies demonstrating that key dimer interface residues directly impact the catalytic activity of 3CL^pro^ (62).

In summary, this study elucidates the critical involvement of the SADS-CoV nsp5 protein in the viral replication process and establishes a theoretical foundation for its potential as a target for antiviral intervention. Although certain limitations exist, these findings provide a valuable framework for the development of future therapeutic strategies aimed at combating SADS-CoV infection.

## MATERIALS AND METHODS

### Cells and viruses

HEK-293T cells (ATCC, CRL-11268), Vero-E6 cells (ATCC, CRL-1586), and ST cells (ATCC, CRL-1746) were maintained in Dulbecco’s modified Eagle medium (DMEM) supplemented with 10% fetal bovine serum (FBS, Gibco, USA) and penicillin–streptomycin at 37°C in a 5% CO_2_ atmosphere. The SADS-CoV strain was previously preserved in our laboratory (47). SADS-CoV was diluted in DMEM supplemented with 10 mg/mL trypsin and inoculated into Vero-E6 cells. After 2 h, the adsorbed SADS-CoV was discarded, and maintenance DMEM containing 10 mg/mL trypsin was added for incubation.

### Sample preparation and RNA-Seq analysis

Vero-E6 cells were seeded in 100 mm dishes (5 × 10^5^ cells/well) and infected with the SADS-CoV strain at an MOI of 1 for 12, 24, and 36 h. Cells treated with the same volume of minimal essential medium for 12, 24, and 36 h were considered mock-infected. After infection, the cells were washed with 1× phosphate-buffered saline (PBS) (Solarbio, China), and total RNA was extracted using the TRIzol reagent (Thermo Fisher Scientific, USA). For SADS-CoV-infected and mock-infected cells, three biological replicates were established per group. Total RNA was isolated and used for RNA-seq analysis. Transcriptome sequencing was conducted by Tsingke Biotechnology Co., Ltd. (Beijing, China). Bioinformatic analysis was performed using the OmicStudio tools.

### Antibodies and reagents

A DYKDDDDK Tag (9A3) mouse mAb (8146, 1:1,000), HA-Tag (C29F4) rabbit mAb (3724, 1:1,000), phospho-IRF-3 (Ser386) rabbit mAb (37829, 1:1,000), phospho-NF-kB P65 (Ser536) rabbit mAb (3033, 1:1,000), anti-mouse IgG(H + L), F(ab′)two fragment (Alexa Fluor 488 Conjugate) (4408, 1:500), anti-rabbit IgG (H + L), and F (ab′) two fragment (Alexa Fluor 594 Conjugate) (8889, 1:500), Cleaved Caspase-9 (Asp353) antibody (9509, 1:1,000), and Phospho-Stat2 (Tyr690) antibody (4441, 1:1,000) were purchased from Cell Signaling Technology. Caspase-8/p43/p18 Polyclonal antibody (13423-1-AP, 1:1,000), GAPDH Monoclonal antibody (60004-1-Ig, 1:1,000), PARP1 Rabbit Polyclonal antibody (13371-1-AP, 1:1,000), IRF3 rabbit polyclonal antibody (11312-1-AP, 1:1,000), STAT1 rabbit polyclonal antibody (10144-2-AP, 1:1,000), GATAD2A Polyclonal antibody (12294-1-AP, 1:1,000), STAT2 rabbit polyclonal antibody (16674-1-AP, 1:1,000), and NF-κB P65 rabbit polyclonal antibody (10745-1-AP, 1:1,000) were purchased from Proteintech. Phospho STAT2 (Tyr690) antibody (AF3342, 1:1,000) was purchased from Affinity. One-step TUNEL *In Situ* Apoptosis Kit (Red, Elab Fluor 594) (Elabscience, China), Taq Pro Universal SYBR qPCR Master Mix, and ClonExpress Ultra One Step Cloning Kit were purchased from Vazyme, Z-DEVD-FMK (TargetMol, China), PF-00835231 (Sigma-Aldrich, USA), PrimeScript RT Master Mix (Perfect Real Time) (Takara, China), FITC Annexin V Apoptosis Detection Kit I (BD Biosciences, USA), Lipofectamine 3000 Transfection Kit (Thermo Fisher, USA), and Pierce Classic Magnetic IP/Co-IP Kit (Thermo Fisher, USA).

### Plasmids and siRNAs

Nsp5 of SADS-CoV was cloned into a pCAGGS-FLAG vector. Full-length cDNA sequences of *Sus scrofa* (pig) GATAD2A (GenBank accession number XM_021083423.1), *Homo sapiens* (human) GATAD2A (GenBank accession number NM_001300946.3), and *Chlorocebus sabaeus* (green monkey) (GenBank accession number XM_007995937.2) were cloned into a pXJ40-HA vector. siRNAs targeting monkey GATAD2A (si-GATAD2A) and negative control siRNA (si-NC) were purchased from Tsingke Biotechnology Co., Ltd. siRNA transfection was performed using Lipofectamine 3000 Transfection Kit reagent, in accordance with the manufacturer’s instructions.

### RNA extraction and RT-qPCR

Cells were washed three times with PBS and lysed with RNAiso according to the manufacturer’s instructions (Takara, China). An amount of 1 μg of total RNA was used to synthesize cDNA by reverse transcriptase (Takara, China). The qRT-PCR by SYBR Premix Ex Taq (Vazyme, China) was conducted according to the manufacturer’s protocol. All primers and siRNA are listed in Table S1. The relative mRNA levels of genes normalized to GAPDH were determined according to the 2^–ΔΔCT^ method.

### Immunofluorescence assay

Nsp5 of SADS-CoV was cloned into a pEGFP-N1 vector. ST cells were transfected with the expression plasmids pEGFP-N1-nsp5 and pEGFP-N1. After 36 h, the cells were fixed with 4% paraformaldehyde (Solarbio, China) for 20 min, washed three times with PBS for 5 min, and then permeabilized with 0.1% Triton X-100 (Beyotime, China) for 15 min at room temperature. After three washes with PBS, the cells were blocked with PBS containing 3% bovine serum albumin (Solarbio, China) for 1 h. Cells were washed with PBS and then incubated with 1:200-diluted anti-GATAD2A mAb at 4°C overnight. After rinsing three times, the cells were incubated with goat anti-rabbit IgG conjugated to fluorescein (1:100 diluted in PBS) (Cell Signaling Technology, USA) for 1 h at room temperature. After washing, cells were stained with DAPI (Beyotime, Shanghai, China) for 5 min at 37°C, and then visualized using confocal microscopy.

### Western blot analysis and Co-IP

Cell lysates were incubated with the selected antibodies at room temperature for 2 h or overnight at 4°C. At room temperature, the antibody complexes were allowed to bind to Protein A/G magnetic beads for 1 h. The magnetic beads were washed twice with IP lysis wash buffer, followed by one wash with pure water. The antibody complexes were eluted. Cell lysates were resolved on 10%–15% SDS-PAGE gels and transferred to polyvinylidene difluoride (PVDF) membranes using a Trans-Blot Turbo transfer system (Bio-Rad). Membranes were blocked with 5% non-fat dry milk in TBST (TBS with 0.1% Tween-20) for 1 h and then probed with the indicated primary antibody in 3% BSA in TBST at 4°C overnight or 2 h at room temperature. After primary antibody incubation, membranes were probed with secondary antibodies in 3% BSA in TBST for 1 h at room temperature on a rocking platform: anti-rabbit or anti-mouse IgG-HRP-conjugated antibody from sheep. Proteins were visualized using ECL and detected by Bio-Rad. Protein band intensity was quantified using ImageJ software.

### Flow cytometry analysis of apoptosis

Vero-E6, ST, and HEK-293T cells seeded in 12-well plates (2 × 10^5^ cells/well) were infected with SADS-CoV or transfected with the SADS-CoV nsp5 and mutant plasmids. Phosphatidylserine exposure was determined by measuring Annexin V binding at the indicated times using an FITC Annexin V Apoptosis Detection Kit (BD Pharmingen), according to the manufacturer’s manual. Cells were washed three times with cold PBS and then were resuspended in 1× Binding Buffer at a concentration of 1× 10^6^ cells/mL. Transfer 100 µL of the solution to a 5 mL culture tube. Then add 5 µL of FITC Annexin V and 5 µL PI. Gently vortex the cells and incubate for 15 min at room temperature in the dark. Add 400 µL of 1× Binding Buffer to each tube. The percentage of apoptotic cells was calculated with a Beckman Coulter DxFLEX flow cytometry system, and the data were analyzed by FlowJo v10.

### Reactive oxygen species assay

The stock solution was diluted to a working concentration of 20 μM with serum-free medium. The ratio of the staining solution to the diluent is 1:250. Cells were cultured in 12-well plates at a density of 5 × 10^5^ cells per well. In total, 400 μL of staining solution at a working concentration of 20 μM was added to each well. The plates were incubated at 37°C in the dark for 30 min, with gentle mixing every 10 min. The staining solution was then removed, and the cells were washed twice with 1× PBS. The percentage of ROS-positive cells was calculated with a Beckman Coulter DxFLEX flow cytometry system, and the data were analyzed by FlowJo v10.

### Structural prediction and molecular docking

The protein models used for docking were GATAD2A (Uniprot ID: Q86YP4) and SADS-CoV nsp5. The HDOCK SERVER (http://hdock.phys.hust.edu.cn/) was employed for the protein-protein molecular docking. GATAD2A was selected as the receptor protein and mKuT6z as the ligand protein. The pre-processing of the proteins was accomplished using PyMol 2.4 (removing water molecules and redundant ligands and adding hydrogen atoms). Docking Score, Confidence Score, and Ligand RMSD were used as the evaluation criteria for the docking. The docking results were set to output the top 10 best docking positions. The model with the highest score was chosen as the best docking model. Finally, the docking results were visualized using Pymol 2.4 software.

### Statistical analysis

Survival curves and analysis were done using Prism 9 for macOS. Data are shown as the mean ± standard deviation (SD) of three independent experiments done in triplicate. Results were analyzed by one-way ANOVA. Differences with *P* < 0.05 were considered significant.

## Data Availability

All data generated or analyzed during this study are included in this published article.

## SUPPLEMENTAL MATERIAL

Supplemental material mbio.00785-26-s0001.docx Fig. S1 to S3; Table S1.

ASM does not own the copyrights to Supplemental Material that may be linked to, or accessed through, an article. The authors have granted ASM a non-exclusive, world-wide license to publish the Supplemental Material files. Please contact the corresponding author directly for reuse.
