# Supplementary material for: Swine acute diarrhea syndrome coronavirus nsp5 induces apoptosis by targeting GATA zinc finger domain-containing protein 2A (GATAD2A/p66α)
Source: mBio. 2026 Jun 15;17(7):e00785-26. doi: 10.1128/mbio.00785-26 (PMC13343927; doi:10.1128/mbio.00785-26)
Supplement: Supplemental material — Fig. S1 to S3; Table S1. [file mbio.00785-26-s0001.docx]

**Supplemental Materials**

**Swine acute diarrhea syndrome coronavirus nsp5** **induces apoptosis by targeting GATA zinc finger domain-containing protein 2A (GATAD2A/p66α)**

Haixin Huang ^1,2,3^, Xin Wang ^4^, Yan Qin ^3^, Yuying Li ^3^, Jiahan Gang ^5^, Xinyu Zhang ^3^, Lulu Xie ^3^, Yimin Zhou ^3^, Qiaoqiong Wang ^3^, Wei Chen ^3^, Yuanze Sun ^3^, Zaijuan Zhang ^3^, Xianfu Ke ^6#^, Tian Lan ^3#^, Wenchao Sun ^3#^

^1^ School of Public Health, Beihua University, Jilin City, Jilin Province, 132013, China.

^2^ College of Veterinary Medicine, Northwest A&F University, Yangling, Shaanxi, 712100, China.

^3^ Wenzhou Key Laboratory for Virology and Immunology, Institute of Virology, Wenzhou University, Wenzhou, Zhejiang, 325035, China.

^4^ Jilin Provincial Center for Disease Control and Prevention, Changchun, Jilin Province, 130062, China.

^5^ College of Animal Science and Veterinary Medicine, Heilongjiang Bayi Agricultural University, Daqing, Harbin, 163319, China.

^6^ Zhejiang Key Laboratory of High-level Biosafety and Biomedical Transformation, Hangzhou Medical College, Hangzhou, Zhejiang, 311305, China.

#Corresponding author:

Associate Prof. Wenchao Sun

Email addresses: [sunwenchao131@163.com](mailto:sunwenchao131@163.com)

Tian Lan

Email addresses: [827002151@qq.com](mailto:827002151@qq.com)

Xianfu Ke

Email: kexfke@ 163.com


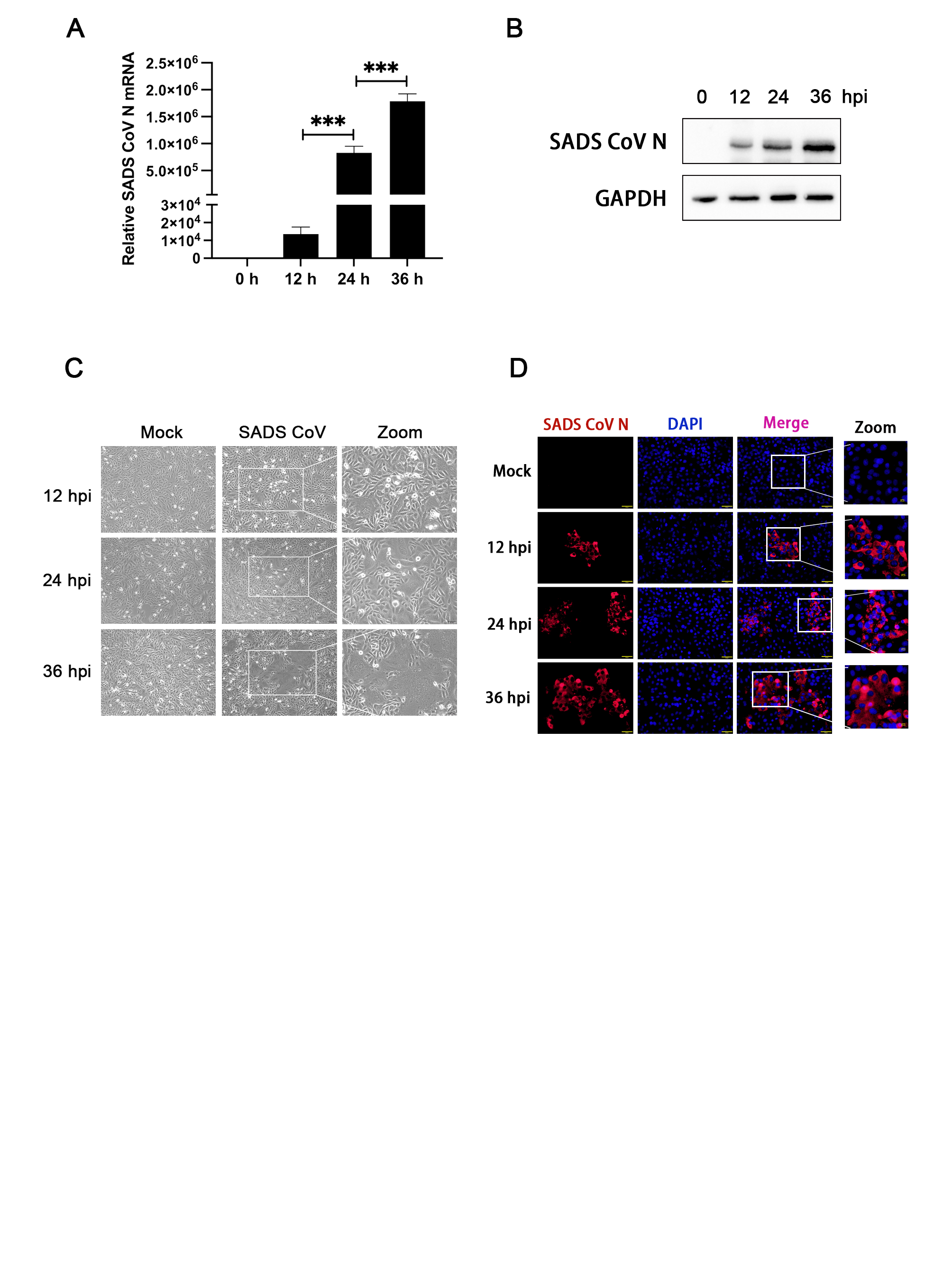


**Figure S1. Replication of SADS-CoV in Vero-E6 cells.**

**(A)** Vero-E6 cells were infected with the SADS-CoV strain (MOI=0.1) and then harvested at 0 h, 12 h, 24 h and 36 h to measure SADS-CoV N mRNA levels by RT-qPCR. (**B)** Vero-E6 cells were infected with the SADS-CoV strain (MOI=0.1) at 0 h, 12 h, 24 h and 36 h cell lysates were analyzed by Western blotting with anti-SADS-CoV N protein antibodies. (**C)** Vero-E6 cells were infected with SADS-CoV strain (MOI=0.1) at 12 h, 24 h and 36 h. **(D)** Vero-E6 cells were infected with SADS-CoV strain (MOI=0.1). Cells were fixed and incubated with rabbit anti-SADS-CoV polyclonal sera (1:200) at 12 h, 24 h and 36 h post infection. Immunofluorescence assays were used to further observe intracellular propagation of SADS-CoV.


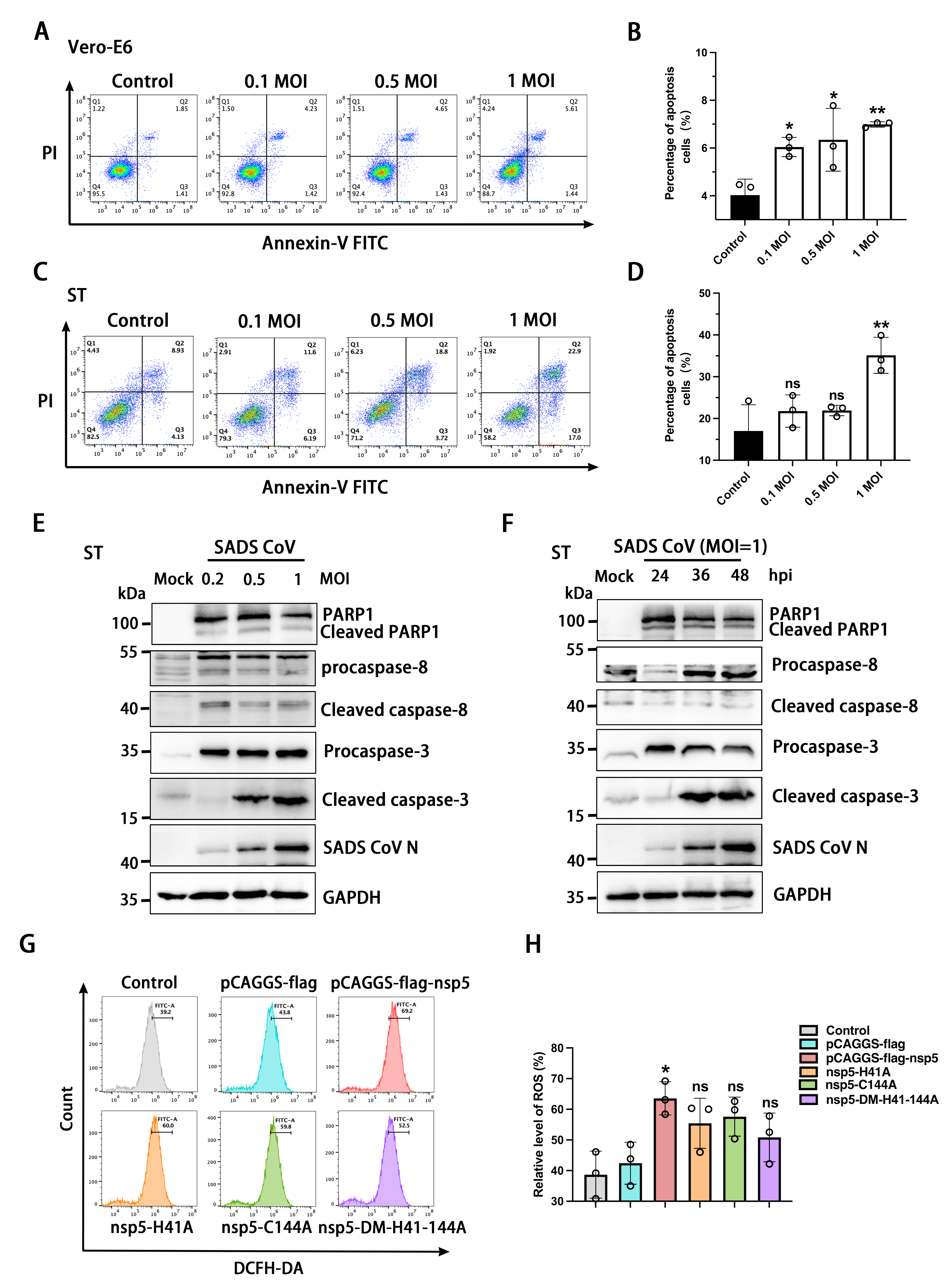


**Figure S2** **SADS-CoV infection induces apoptosis *in vitro*.**

**(A)** Flow cytometry analysis of apoptotic cells in Vero-E6 cells infected with SADS-CoV at different MOIs. Mock or virus-infected cells collected at 36 h were subjected to dual Annexin V and PI labeling and analyzed by FACS. (**B)** Percentage of apoptotic cells of Vero-E6 cells infected with SADS-CoV at different MOIs. **(C)** Flow cytometry analysis of apoptotic cells in ST cells infected with SADS-CoV at different MOIs. Mock or virus-infected cells collected at 36 h were subjected to dual Annexin V and PI labeling and analyzed by FACS. (**D)** Percentage of apoptotic cells of ST cells infected with SADS-CoV at different MOIs. **(E-F)** SADS-CoV infection activates caspase-8, -3 and cleaved PARP *in vitro*. Western blot analysis of caspase activation in SADS-CoV-infected cells (MOI=1) at different times or MOIs at 36 h. (**G)** HEK-293T cells were transfected with pCAGGS-flag empty vector, pCAGGS-flag-nsp5 expressing plasmid, mutant expressing plasmids H41A , C144A and H41-144A, respectively, and collected at 36 h after transfection. Then cells were subjected to reactive oxygen species assay by FACS. (**H)** Percentage of ROS-positive cells of HEK-293T cells transfected with pCAGGS-flag, pCAGGS-flag-nsp5, mutant expressing plasmids H41A, C144A and H41-144A.


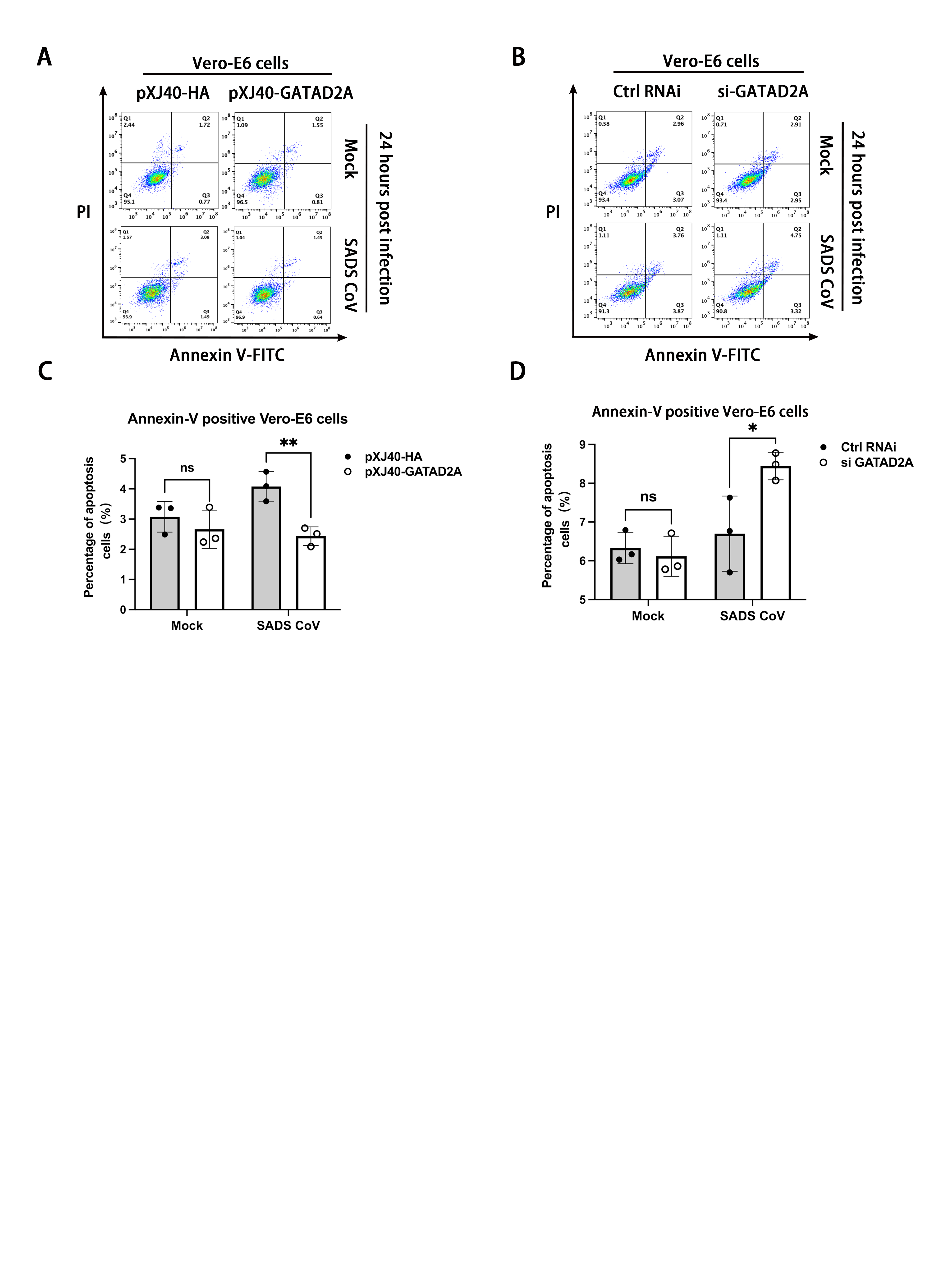


**Figure S3 GATAD2A inhibits SADS-CoV-induced apoptosis.**

(**A)** pXJ40-HA and pXJ40-mGATAD2A were transfected into Vero-E6 cells. After 24 h, then infected with SADS-CoV for 24 h, then subjected to dual Annexin V and PI labeling and analyzed by FACS. (**B)** Vero-E6 cells were transfected with negative control siRNA and GATAD2A siRNA. After 24 h, then infected with SADS-CoV for 24 h, then cells were subjected to dual Annexin V and PI labeling and analyzed by FACS. **(C)** Percentage of apoptosis analysis with Vero-E6 cells transfected pXJ40-HA and pXJ40-mGATAD2A, then infected with SADS-CoV for 24 h. **(D)** Percentage of apoptosis analysis with Vero-E6 cells transfected negative control siRNA and GATAD2A siRNA, then infected with SADS-CoV for 24 h.

| Primer | Sequence（5′—3′） |
| --- | --- |
| pCAGGS-SARS-CoV-nsp5-F | CGAGCTCGCGGCCGCGGTACCAGTGGTTTTAGGAAAATGGCATTC |
| pCAGGS-SARS-CoV-nsp5-R | CTTGTAATCACCTCCCTCGAGTTGGAAGGTAACACCAGAGCATT |
| pCAGGS-SARS-CoV-2-nsp5-F | CGAGCTCGCGGCCGCGGTACCAGTGGTTTTAGAAAAATGGCATTCC |
| pCAGGS-SARS-CoV-2-nsp5-R | CTTGTAATCACCTCCCTCGAGTTGGAAAGTAACACCTGAGCATTG |
| pCAGGS -PEDV-nsp5-F | CGAGCTCGCGGCCGCGGTACCGCTGGCTTGCGTAAGATGGC |
| pCAGGS-PEDV-nsp5-R | CTTGTAATCACCTCCCTCGAGCTGAAGATTAACGCCATACATTTGA |
| pCAGGS-MERS-CoV-nsp5-F | CGAGCTCGCGGCCGCGGTACCAGCGGTTTGGTGAAAATGTCA |
| pCAGGS-MERS-CoV-nsp5-R | CTTGTAATCACCTCCCTCGAGCTGCATAACCACACCCATAATCTG |
| p-GATA-506-F | ACTGAAGGCCGTCATAAAACCGCGGCGTAAGT |
| p-GATA-506-R | TTATGACGGCCTTCAGTGCGGGGTGTGGGGCG |
| p-GATA-531-F | TGTGCTAGCCGCCTCCAGCCAGCTGTCCCGGG |
| p-GATA-531-R | TGGAGGCGGCTAGCACAGCCCCGTTACTCCAG |
| p-GATA-535-F | TACAGGCCTCCAGCGCCCTGTCCCGGGGCTCCGCC |
| p-GATA-535-R | GGCGCTGGAGGCCTGTAGCACAGCCCCGTTAC |
| p-GATA-558-F | CAAACTGGCCAATGCAGCCTCGGCCACAGCCC |
| p-GATA-558-R | CTGCATTGGCCAGTTTGGGTGACTGGCTGAAC |
| SARS-CoV-2-nsp5-41F | TCCAAGAGCCGTGATCTGCACCTCTGAAGACATG |
| SARS-CoV-2-nsp5-41R | AGATCACGGCTCTTGGACAGTAAACTACGTCATCAA |
| SARS-CoV-2-nsp5-145F | ATGGTTCAGCCGGTAGTGTTGGTTTTAACATAGATTATG |
| SARS-CoV-2-nsp5-145R | ACTACCGGCTGAACCATTAAGGAATGAACCCTT |
| MERS-CoV-nsp5-41F | CCCACGAGCCGTAATGTGCCCGGCTGACCAGT |
| MERS-CoV-nsp5-41R | ACATTACGGCTCGTGGGCACCAGACTGTGTTG |
| MERS-CoV-nsp5-148F | GTTCTGCCGGTAGTGTTGGTTACACCAAGGAGG |
| MERS-CoV-nsp5-148R | AACACTACCGGCAGAACCACACAGAAAGGAACCCT |
| p-GATA-485-F | TCCTACAGGCCGGCGCTGCCCCTACGCAGGCC |
| p-GATA-485-R | AGCGCCGGCCTGTAGGAGGCGCTGCTCAATCT |
| p-GATA-491-F | CCCTACGGCCGCCAAGGCGGAGCCTGCTGCCG |
| p-GATA-491-R | CCTTGGCGGCCGTAGGGGCAGCGCCCTGCTGT |
| p-GATA-553-R | TTGGGTGAGGCGCTGAACGTGTGCAGGATGCC |
| p-GATA-1-568-R | GCTTTAATAAGATCTGGTACCCTGACAAGGGCTGTGGCC |
| PDCoV nsp5-144F | TCCTGAACGGCGCCGCCGGCAGCGTGGGGTACACA |
| PDCoV nsp5-144R | GGCGGCGCCGTTCAGGAAGCTGGCGTAGATCA |
| PDCoV nsp5-41F | GCCGTGATCGGCAAGTTCAGGGGCGATCAGTG |
| PDCoV nsp5-41R | AACTTGCCGATCACGGCCCTTGGGCAGTACACCACGT |
| h-IFN-β-F | TCTTTCCATGAGCTACAACTTGCT |
| h-IFN-β-R | GCAGTATTCAAGCCTCCCATTC |

**Table S1 Sequences of the primers and siRNAs**

**Continued table S1 Sequences of primers**

| Primer | Sequence（5′—3′） |
| --- | --- |
| h-RIG-I-F | TTCCCAGACCACAGGAATACC |
| h-RIG-I-R | GCAGGAGAACAAAGCCCAACT |
| h-MDA-5-F | TTCCGCTATCTCATCTCGTGC |
| h-MDA-5-R | ACTGTCCTCTGAATCTGCTCC |
| h-MAVs-F | GCCCATCAACTCAACCCGTG |
| h-MAVs-R | TCCTCATTTCTGCTGCTCCC |
| h-ISG54-F | CACCTCTGGACTGGCAATAGC |
| h-ISG54-R | GTCAGGATTCAGCCGAATGG |
| h-ISG56-F | GCTTTCAAATCCCTTCCGCTAT |
| h-ISG56-R | GCCTTGGCCCGTTCATAAT |
| h-IFIT3-F | TCAGAAGTCTAGTCACTTGGGG |
| h-IFIT3-R | ACACCTTCGCCCTTTCATTTC |
| h-IFIT5-F | GGCCAAAATAAAGACGCCCTT |
| h-IFIT5-R | GACCAGGCTTCGTACTTCTTC |
| h-IRF3-F | AGAGGCTCGTGATGGTCAAG |
| h-IRF3-R | AGGTCCACAGTATTCTCCAGG |
| h-IRF9-F | GCTGGTGGAGAACTCAAGGCT |
| h-IRF9-R | GAGGTCAGGGAAGAGGGAAAG |
| h-NF-κB-F | TGGACCGCTTGGGTAACTCT |
| h-NF-κB-R | CCACCAGCAGCAGCAAACAT |
| h-GAPDH-F | TCTGCTCCTCCTGTTCGACAG |
| h-GAPDH-R | CCCAATACGACCAAATCCGTT |
| SADS-CoV N-F | AACGGATTCAGGGTGTGCAT |
| SADS-CoV N-R | CGATTGCGAACACCAAGACC |
| m-GAPDH-F | GTGAAGGCTGAGAACGGGAA |
| m-GAPDH-R | AAATGAGCCCCAGCCTTCTC |
| m-GATAD2A-F | ACCCGATGTGATTGTGCTGT |
| m-GATAD2A-R | CTTTTCATGAGGGCCTCGGT |
| si-monkey-GATAD2A-sense | GGAUGAUCAAACAGCUGAA |
| si-monkey-GATAD2A-antisense | UUCAGCUGUUUGAUCAUCC |
|  |  |
